# Single locus complementary sex determination in Hymenoptera: an "unintelligent" design?

**DOI:** 10.1186/1742-9994-3-1

**Published:** 2006-01-05

**Authors:** Ellen van Wilgenburg, Gerard Driessen, Leo W Beukeboom

**Affiliations:** 1 Department of Zoology, University of Melbourne, VIC 3010 Australia.; 2 Department of Animal Ecology, Institute of Ecological Science, Vrije Universiteit, De Boelelaan 1085, 1081 HV Amsterdam, The Netherlands; 3 Evolutionary Genetics, Centre for Ecological and Evolutionary Studies, University of Groningen, P.O. Box 14, NL-9750 AA Haren, The Netherlands; 4 Institute of Biology Leiden, University of Leiden, P.O. Box 9516, NL-2300 RA Leiden, The Netherlands

## Abstract

The haplodiploid sex determining mechanism in Hymenoptera (males are haploid, females are diploid) has played an important role in the evolution of this insect order. In Hymenoptera sex is usually determined by a single locus, heterozygotes are female and hemizygotes are male. Under inbreeding, homozygous diploid and sterile males occur which form a genetic burden for a population. We review life history and genetical traits that may overcome the disadvantages of single locus complementary sex determination (sl-CSD). Behavioural adaptations to avoid matings between relatives include active dispersal from natal patches and mating preferences for non-relatives. In non-social species, temporal and spatial segregation of male and female offspring reduces the burden of sl-CSD. In social species, diploid males are produced at the expense of workers and female reproductives. In some social species, diploid males and diploid male producing queens are killed by workers. Diploid male production may have played a role in the evolution or maintenance of polygyny (multiple queens) and polyandry (multiple mating). Some forms of thelytoky (parthenogenetic female production) increase homozygosity and are therefore incompatible with sl-CSD. We discuss a number of hypothetical adaptations to sl-CSD which should be considered in future studies of this insect order.

## Introduction

The insect order Hymenoptera comprises over 200,000 species of ants, bees, wasps and sawflies. All members have haplodiploid sex determination; males are haploid (one chromosome set) and females are diploid (two chromosome sets). Arrhenotoky is the most common mode of reproduction; males develop parthenogenetically from unfertilised eggs and females from fertilised eggs. Arrhenotokous females typically have control over fertilisation by releasing sperm to an egg upon oviposition, and can facultatively adjust the sex ratio of their progeny. In thelytokous species diploid females develop parthenogenetically from unfertilised eggs and there are no males [1,2]. Thelytoky has independently arisen in several groups [3].

Sex determination in haplodiploids involves no heteromorphic sex chromosomes, thus the only difference between males and females is the number of chromosome sets. Several different genetic mechanisms of sex determination occur in Hymenoptera. One mechanism that has been known for over 60 years is single locus complementary sex determination (sl-CSD, [4,5]). Under sl-CSD, the sex of an individual depends on the allelic composition at a single locus. Hemizygous haploid individuals are male and diploid individuals are female when heterozygous, but male when homozygous. Thus, in contrast to the standard arrhenotokous situation of haploid males from unfertilized eggs, some males can be diploid and those males are of biparental origin. These males are typically sterile [6,7] and sometimes have reduced viability [5,8-10]. In addition, they can produce diploid sperm which leads to triploid (sterile) offspring [6]. In a number of cases diploid males can be morphologically distinguished by their size, weight or the density of wing microchaetae.

The *csd *gene has recently been cloned and sequenced from the honey bee [11], but its exact mode of action in sex determination is not yet understood [12]. Several attempts to isolate the *csd *gene from other Hymenoptera have to date been unsuccessful. Very little is known about the genetic regulation of sex determination in species without CSD. Although several models have been proposed, they have little empirical support [3,13,14]). In this paper, we will not review the existing evidence for these models again, but instead we consider a number of life history and genetical aspects that are relevant to single locus complementary sex determination (sl-CSD).

Diploid male production (DMP) in Hymenoptera may have a number of important evolutionary consequences. Many authors have considered one or more aspects of DMP for the population dynamics, including colonisation ability [15], population growth [16-20], sex allocation and mating structures [8,21,22]; the evolution of eusociality [23]; the evolution of thelytoky [3,24,25], and the application of parasitoids in biological control [7]. The most complete overview of the consequences of CSD has been given by Cook and Crozier [8].

It is generally accepted that haplodiploids are less affected by the deleterious effects of inbreeding since recessive mutations are more effectively expelled from the population through haploid males [26-29]. However, sl-CSD can be particularly detrimental under inbreeding conditions because it produces proportionally more homozygotes (diploid males) than under outbreeding. Under sl-CSD, matched matings (i.e. when the female and male partners share a similar sex allele [30]) result in broods in which 50 percent of fertilised eggs develop into diploid males. Sib-matings increase the chance of such matched matings. Because diploid males are often infertile or less viable they impose a genetic load on populations. Hence, there will be frequency-dependent selection on sex alleles where rare alleles in a population will have a selective advantage. The number of alleles at the sex locus has been reported to vary from 9 up to 86 [8]. In a random mating population, the probability of matched mating (defined as a mating between individuals carrying an identical sex allele) will be 2/k [8], where k is the effective number of sex alleles. A proportion of 1/k of the diploid individuals are thus expected to be homozygous males. In recent years more species of different groups have been investigated for the presence or absence of sl-CSD. In addition, since Cook and Crozier's [8] overview, several authors have addressed one or more consequences of sl-CSD for the biology of different hymenopteran groups. Moreover, some of the commonly accepted consequences of sl-CSD were recently challenged by a new study of Cowan and Stahlhut [31]. In this paper we collate the new data. The purposes of our paper are: (1) to extend and update previous overviews on the distribution of sl-CSD in the Hymenoptera, (2) to consider some life history traits that may reduce the load of DMP, (3) to explore what alternative mechanisms, both at the genetic and population biological level, may have evolved to minimise the costs of DMP and finally (4) to point out a number of priority research topics that are, in our opinion, crucial for a full understanding of the many genetical, ecological and evolutionary aspects of sl-CSD. We hope that this review will highlight the key areas of contention in this topic, and stimulates further research.

## Taxonomic distribution of CSD

Biologists have used different types of evidence for the presence of sl-CSD. In increasing order of confidence level they can be ranked as follows: 1) post-hoc explanations for exceptionally high-male biased sex ratios in cultures or field surveys, 2) on the basis of the verification of male diploidy through cytological (chromosome number), morphological (size, weight, density of wing microchaetae), genetical (microsatellites) or electrophoretical (allozymes) methods, 3) on the basis of sex ratios in inbreeding experiments that are in accordance with predictions under sl-CSD, 4) a combination of 2 and 3, and 5) linkage mapping of the sex locus and/or its molecular characterization. sl-CSD has now been demonstrated in over 60 species of Hymenoptera, including sawflies (Symphyta), parasitoid wasps (Apocrita; Parasitica), and ants, bees and wasps (Apocrita; Aculeata) [3,7,8,32,33]. Tables 1 and 2 summarise the non-social and social Hymenoptera respectively for which sl-CSD has been supposed to be present. They expand the list of species published by Stouthamer *et al*. [7], Cook [3] and Periquet *et al*. [34] by two-fold, but all added species belong to previously investigated groups.

**Table 1:** Non-social hymenopteran species for which sl-CSD has been proposed to be the sex determining mechanism. Confidence codes indicate the levels of evidence: 1 = post hoc explanations for exceptionally high male biased sex ratios in cultures or field surveys, 2 = on the basis of the verification of male diploidy through cytological (chromosome number), morphological (size, weight, density of wing microchaetae), genetical (microsatellites) or electrophoretical (allozymes) methods, 3 = on the basis of the sex ratios in inbreeding experiments in accordance with predictions under CSD, 4 = the joint combination of level 2 and 3, and 5 = linkage mapping of the sex locus and/or its molecular characterization

Species	Confidence code	Reference
**Sub-order Symphyta**		
**Family Tenthredinideae**		
*Athalia rosae*	4	[117]
**Family Diprionidae**		
*Neodiprion nigroscutum*	4	[118]
*Neodiprion pinetum*	2	Wallace pers. comm. in [7]
**Sub-order Apocrita Infra-order Parasitica**		
**Family Braconidae**		
*Aphidius rhoplosiphi*	3	[119]
*Bracon brevicornis*	4	[120]
*Bracon hebetor*	4	[121]
*Bracon serinopae*	4	[122]
*Cotesia rubecula*	2	Steiner pers. comm. in [7]
*Cotesia glomerata*	3	[20]
*Microplitis croceiceps*	4	[123]
**Family Ichneumonidae**		
*Bathyplectes curculionis*	2	[124]
*Diadegma armillata*	4	[32]
*Diadegma chrysostictos*	4	[125]
*Diadegma eucerophaga*	4	[32]
*Diadegma fabriciane*	4	[32]
*Diadegma fenestralis*	4	[32]
*Diadegma insulare*	4	[32]
*Diadegma pulchellus*	4	[32]
*Diadegma semiclausum*	4	[126]
*Diadromus pulchellus*	4	[34,127]
*Heteropelma scaposum*	1	[128]
*Venturia canescens*	3	[62]
**Sub-order Apocrita Infra-order Aculeata**		
**Family Vespidae**		
*Ancistrocerus antilope*	2	[58]
*Euodynerus foraminatus*	4	[56,57]

**Table 2:** Social hymenopteran species for which sl-CSD has been proposed to be the sex determining mechanism. Confidence codes are as explained in Table 1.

Species	Confidence code	Reference
**Sub-order Apocrita infra-order Aculeata**		
**Family Apidae**		
*Andrena scotica*	2	[111]
*Apis cerana*	4	[68,75,129,130]
*Apis mellifera*	5	[68,130,131,132,133,134]
*Augochlorella striata*	2	[135,136]
*Bombus atratus*	4	[17,137,138,139]
*Bombus terrestris*	5	[68,140,141,142,143,144]
*Euglossa tridentata*	2	[23]
*Euglossa meriana*	2	[23]
*Euglossa imperialis*	2	[23]
*Euglossa sapphirina*	2	[23]
*Halictus poeyi*	2	[63]
*Lasioglossum zephyrum*	2	[71,145]
*Melipona compressipes*	2	[68,146]
*Melipona quadrifasciata*	4	[68,147,148]
*Scaptotrigona postica*	2	[149]
*Trigona carbonaria*	2	[150]
*Trigona quadrangula*	2	[151]
**Family Vespidae**		
*Liostenogaster flavolineata*	2	[59]
*Mischocyttarus immarginatus*	2	J. Strassmann pers. comm. in [65]
*Polistes apachus*	2	[152]
*Polistes chinensis antennalis*	2	[153]
*Polybioides tabidus*	2	[76]
*Vespa crabro*	2	[137]
**Family Formicidae**		
*Acromyrmex heyeri*	2	[154]
*Acromyrmex striatus*	2	[154]
*Lepthotorax kutteri*	2	[69,155,156]
*Myrmoxenus stumperi*	2	[86,156]
*Formica aquilona*	2	[60]
*Formica lugubris*	2	[60]
*Formica polyctena*	2	[60]
*Formica pressilabris*	2	[142,143]
*Formica truncorum*	2	[60]
*Formica rufa*	2	[60]
*Harpagoxenus sublaevis*	2	[156,157]
*Lasius sakagamii*	2	[158]
*Leptothorax acervorum*	2	[159]
*Leptothorax muscorum*	2	[160,161]
*Leptothorax nylanderi*	2	[162]
*Proformica longiseta*	2	[163]
*Pseudolasius emeryi*	2	[164]
*Rhytidoponera chalybaea*	2	[165,166]
*Rhytidoponera confusa*	2	[165,166]
*Solenopsis invicta*	2	[18,164,167,168]

The presence of members with sl-CSD in each major hymenopteran subgroup has led to the suggestion that sl-CSD is the ancestral mode of sex determination in the Hymenoptera [3,35]. However, this conclusion seems premature since our knowledge of the phylogenetic distribution of sl-CSD is still incomplete. Some recent studies in the parasitoid family Braconidae show that sl-CSD occurs in particular subfamilies while it is absent in closely related ones (compare Tables 1 and 3). Even more striking is the presence of species with and without sl-CSD within one genus: *Cotesia*[7,20]. This suggests that shifts between sl-CSD and alternative mode(s) of sex determination may easily occur [36-38]. Another notable conclusion from comparing Tables 1 and 3 is the apparent absence of non-CSD species in the social Hymenoptera (see also below). Clearly, there is a need for further testing in the Hymenoptera before general conclusions can be made about the phylogenetic distribution of sl-CSD.

**Table 3:** Species in which sl-CSD is shown to be absent

Species	Reference
**Sub-order Apocrita Infra-order Parasitica**	
**Family Cynipidae**	
*Diplolepis rosae*^1^	[99]
*Diplolepis spinosissimae*	[98]
**Family Figitidae**	
*Leptopilina boulardi*	[169]
*Leptopilina heterotoma*	[170]
**Family Chalcididea**	
*Dinarmus vagabundus*	[171]
*Melittobia chalybii*	[172]
*Melittobia spp.*	[35]
*Muscidifurax raptor*	[98,173,174]
*Muscidifurax zaraptor*	[174]
*Nasonia vitripennis*^1^	[108]
*Trichogramma spp.*	[89]
**Family Braconidae**	
*Asobara tabida*	[36]
*Alysia manducator*	[36]
*Cotesia flavipes*	[37]
*Cotesia sesamiae*	[37]
*Heterospilus prosopidis*	[38]
**Family Scelionidae**	
*Telonomus fariae*	[175]
**Sub-order Apocrita infra-order Aculeata**	
**Family Bethylidae**	
*Goniozus nephanditis*	[33]

^1 ^For *Nasonia vitripennis *[40] and *Diplolepis rosae *[99] uniparental diploid males have been found that apparently arose by mutation

Importantly, there are some situations where the relation between diploid males and CSD is unclear. For example, diploid males have been reported from hybridization of two subspecies of fig wasps [39]. In *Nasonia vitripennis *diploid males have been found to occur spontaneously in laboratory cultures [40,41]. They are not the result of sl-CSD, are fully fertile and produce diploid sperm. These cases fall outside the scope of this paper.

## Life history aspects of CSD

### Non Social Hymenoptera

Table 1 summarizes the non-social Hymenoptera in which sl-CSD is proposed to be the sex determining mechanism. Since many different methods have been used to infer the presence of sl-CSD we have graded all the species according to the five categories of increasing confidence level of evidence that were distinguished in the previous section. Apart from parent-offspring matings, which are probably extremely rare in nature, the highest risk of producing diploid males is in sib matings. Mating in gregarious parasitoid species (*i*.*e*. two or more offspring emerging from one host) generally occurs among individuals emerging from a single host before the females disperse [42]. Gregariousness may therefore be in conflict with sl-CSD. The three *Bracon *species in Table 1 seem to violate this prediction. In *B. hebetor *sex ratios have been reported to be female-biased [21,43]. Nevertheless, sib mating in *B. hebetor *is rare for a number of reasons. Females exhibit a pre-mating refractory period during which dispersal takes place [44-46], they have a mating preference for males that emerged from a different host [45], and males aggregate in leks that attract sperm-depleted females. A pre-mating refractory period of 4 to 5 hours after emergence has also been found in *B. brevicornis *[47]. In the only other gregarious species in Table 1, *Cotesia glomerata*, 50 to 100 per cent of the females and, approximately, 30 per cent of the males disperse immediately after emergence from their natal patch. This results in only a minority of 25 per cent of females mating with sib males in the field [20]. Clearly, these behaviours promote an outcrossing mating system.

In solitary parasitoid species (*i*.*e*. only one offspring emerging from a host) and sawflies the probability of sibs meeting each other in the field will depend on their temporal and spatial distribution. Oviposition in solitary species is a sequential process, where the searching time for oviposition sites or hosts causes a time delay between successive ovipositions. Additionally, differences in quality of sites and hosts and differences in microclimate induce further desynchronisation of development and emergence time. This, together with the fact that all solitary species in Table 1 are good dispersers with both sexes fully winged, may contribute considerably to an outbreeding mating structure. However, apart from these general mechanisms inherent to the solitary life cycle, there may be other aspects that further reduce the probability of sib mating.

Some species in Table 1 like *Athalia rosae *and *Microplitis croceiceps *initially produce female biased sex ratios, but lay male biased sex ratios later on in life [48,49], causing a further temporal segregation of female and male sibs. A similar effect results from the tendency of *Diadegma *species to lay male eggs in young, small hosts and female eggs in older and larger hosts [50,51]. Other species in Table 1 divide their total egg complement over many host patches thereby creating a spatial segregation of offspring. In *Neodiprion nigroscutum*, *Bathyplectes curculionis *and *Diadromus pulchellus*, for example, this results from the fact that their hosts occur in low numbers per patch [52-54]. Comparing the behaviour of thelytokous and arrhenotokous forms of *V. canescens *Thiel *et. al. *[55] recently found strong indications that the oviposition behaviour of the arrhenotokous form is specifically adapted to promote outbreeding.

Similarly to gregarious parasitoids, the offspring of nest building wasps also are likely to meet sibs early in life. Females of the hunting wasp *Euodynerus foraminatus *build nests in which they store prey and lay eggs. Males develop faster than females and up to 66 per cent of the females in a nest mate with their brothers [56,57]; this species undoubtedly has sl-CSD. However, Cowan and Stahlhut [31] recently have shown that the diploid males in *E. foraminatus *are normally fertile and able to transmit their genes to their daughters. It seems that diploidy does not entail many costs in these males. This case appears unique, but shows that one needs to be cautious in generalising that diploid males produced by CSD are an evolutionary dead end. For another cavity nesting Vespid, the potter wasp *Ancistrocerus antilope*, the other Vespoidea in Table 1, extremely high levels (>90 per cent) of inbreeding have been found in natural populations [58]. The same study also reported that around 25 per cent of the males collected from trap nests in the field were diploid. If CSD is the mechanism causing this diploidy, the two findings could point to a similar 'immunity for male diploidy' as in *E. foraminatus*.

It must be emphasised that for none of the solitary species in Table 1 life histories or mating and oviposition behaviour have been studied with special reference to CSD. For example, little is known about avoidance of sib mating in solitary parasitoid species in general, simply because it remains relatively uninvestigated. Moreover, although the mechanisms promoting sib mating avoidance in the gregarious *Bracon *case are likely adaptations to CSD, the aforementioned mechanisms that contribute to temporal or spatial segregation of sibs in solitary species are not necessarily specific adaptations to CSD. Laying small clutches, for example, may serve as a bet-hedging strategy in the first place, while laying male eggs in small hosts a matter of optimal host use. The *Cotesia *genus, in which species with and without CSD occur, could provide a good system to study the adaptive significance of life history traits and behaviour with respect to CSD. The example of *E. foraminatus *shows that asking if and how CSD species reduce the probability of matched matings may lead to surprising new findings.

### Social Hymenoptera

Diploid males have now been detected in more than 40 species of ants, bees and social wasps. Although this study doubles the number of cases compared to previous reviews [3,7,34], contrary to non-social Hymenoptera, sl-CSD has been confirmed only for a small number of social Hymenoptera (Table 2). This may be attributed to difficulties of breeding social hymenopterans in the laboratory. The proportion of diploid males that have been found among the progeny of social Hymenoptera can be remarkably high, indicating either high levels of inbreeding or small variation in the sex determination locus. For the primitively social wasp *Liostenogaster flavolineata*, for example, Strassmann *et al*. [59] found 11 of 71 males to be diploid and in some *Formica *ant species (*F. aquilona*, *F. rufa *and *F. polyctena*) 10 per cent of all males are diploid [60]. In the primitive eusocial bee *Hallictus poeyi *proportions of diploids that are male are estimated to range from 9.1 to 50 per cent [61]. Populations may differ significantly in their DMP. For example, Roubic *et al. *[23] found that, within populations of the colonial genera *Euglossa *and *Eulaema *of Euglossine bees in Panama, an estimated 12–100 per cent of all males are diploid, yet Takahashi *et al. *[62] found almost no diploid males within Brazilian populations of Euglossine bees [63].

In social hymenoptera, diploid males are produced at the expense of workers or female reproductives and are therefore expected to impose severe disadvantages for colony growth and survival. Plowright and Pallett [17], for example, found that colonies of the bumble bee *Bombus atratus *in which 50 per cent of the diploid progeny were male grew significantly slower than colonies producing only workers. Also, incipient monogynous colonies (bearing a single reproductive queen) of the fire ant *Solenopsis invicta *with DMP have a significantly slower colony growth and exhibit higher mortality than those that do not produce diploid males [18]. While diploid males are often sterile, diploid males of *Polistes dominules *wasps are capable of mating and produce triploid offspring. In this species DMP may thus result in a delayed fitness cost for two generations [64].

A number of traits appear to have evolved in social Hymenoptera that reduce the risk of sib-mating. Additionally, there are several other traits that may diminish the costs of DMP. In the following section an overview of these traits is presented for various species known to produce diploid males.

#### Avoidance of sib-matings

Social Hymenoptera show several behavioural and morphological traits that reduce the probability of mating amongst siblings and most species have inbreeding levels not significantly different from zero [65]. Most species avoid inbreeding by dispersal of both sexes, and males and females will often leave the nest at different times [66,67]. Both sexes of *Apis *and *Melipona *bees, for example, are known to fly great distances in order to mate in population-wide mating swarms [68]. Alternative sexual dispersal behaviour is found in the ants *Harpagoxenus sublaevis *and *Doronomyrmex kutteri*. In these species, the males leave their natal nest and disperse to find unmated queens, while the females walk only a short way from the nest to exhibit a so-called "female calling" behaviour to attract mates [69,70]. Workers of a number of *Bombus *species reduce the risk of inbreeding by actively removing young males from the colony, thereby preventing them from mating with their own sisters. Males are attacked when they are 4–5 days old and eventually killed if they do not leave the colony [17]. In the primitively social bee *Lasioglossum zephyrum*, the male bees recognise and avoid mating female kin through olfactory signals [71].

#### Removal of diploid male larvae

Some social Hymenoptera remove diploid males in an early stage, thereby avoiding rearing costs [72]. Woyke [73] showed that diploid male larvae of the honeybee *Apis mellifera *are removed and cannibalised almost immediately after hatching. The hydrocarbon patterns of diploid male larvae of *A. mellifera *differ from those of diploid worker and haploid drone larvae and may be used by workers to distinguish between the three types of larvae [74]. The diploid males of another honeybee, *A. cerana *are also removed, one day after hatching [75]. In the African swarm-founding wasp *Polybiodes tabidus *and *Formica *ants, diploid males are only detected at times when the colony produces sexual offspring, suggesting that in non-sexual brood males are eliminated at early developmental stages [60,76].

#### Removal of diploid male-producing queens

In contrast with the larvae of the honeybee, which are situated in open cells, the larvae of *Melipona *bees are reared in sealed cells. *Melipona *bees are therefore not able to detect and remove diploid males. Diploid males of *M. quadrifasciata *have normal survival as immatures [77]. However, when a *M. quadrifasciata *queen produces diploid males, the workers kill the queen and rear a replacement [78].

#### Polyandry and polygyny

If all queens in a population of social Hymenoptera are singly mated, under random mating, a number of females within the population will mate with a male sharing their sex allele and produce diploid progeny of which half will be males. If females mate with multiple males, more females within the population will produce diploid males, but the proportion of males among the diploid progeny per female will be lower. Thus, in polyandrous populations, although the absolute proportion of diploid males will be the same, the variance in DMP among colonies is reduced [79].

The load hypothesis predicts that the load of diploid males will select for monandry or polyandry depending on the relationship between DMP and female fitness [16,78-80]. In case of a linear relationship between DMP and female fitness, sl-CSD in not expected to select for polyandry, but under some non-linear relationships it may. Antolin and colleagues [22], for example, showed theoretically that multiple mating reduces genetic load if populations contain only few sex alleles. In social hymenoptera several factors influencing the relationship between DMP and a queen's fitness, like the timing of the removal of diploid males [78] and the timing of sexual production during colony growth [16] have been suggested to promote polyandry. The load hypothesis predicts selection for polyandry when, for example, colonies of social Hymenoptera can tolerate moderate, but not high frequencies of diploid males, because high levels of diploid males would almost always result in the death of a colony. As a result, the fitness of multiple mated queens within colonies that produce, for example, 25 per cent diploid males could be higher than the average of single mated queens producing 50 per cent or 0 per cent diploid males. At this moment there is, however, no empirical evidence that polyandry has specifically evolved in response to DMP.

In polygynous colonies, the DMP by some queens can be buffered by the presence of workers produced by other queens in the nest. In addition, polygynous colonies often reproduce by fission or budding, and may therefore skip the vulnerable early exponential phase of colony growth, in which the load of diploid males might be fatal [16]. Around 1940, the fire ant *Solenopsis invicta *was introduced from South-America to North-America; diploid males are far more common in the introduced population than in the native populations, probably due to loss of sex alleles [15,81]. In the introduced range, several polygynous populations have apparently evolved independently in only a few decades from the originally monogynous founder population. While diploid males are very common in polygynous colonies, they are absent in monogynous colonies [18]. Ross and Fletcher [18] showed that monogynous colonies which adopted queens rear diploid males in the laboratory and the absence of diploid males in monogynous colonies in the field can thus not be explained by elimination of diploid males at early stages. This suggests that monogynous incipient colonies of *S. invicta *producing diploid males do not survive [82]. While DMP producing queens are likely to benefit greatly from joining a multi-queen colony, it is unclear what role DMP has had in the evolution or maintenance of polygyny in the imported fire ant [18,81].

The load hypothesis predicts an association between monogyny and monandry when colonies with moderate frequencies of diploid males have high mortality [60]. Pamilo *et al *[60] investigated this hypothesis in several *Formica *ant species. In accordance with the theory up to 10 per cent of all males are diploid in species of *Formica *ant with highly polygynous colonies (*F. aquilona*, *F. truncorum *and *F. polyctena*), while no diploid males were found in two mainly monandrous/monogynous species (*F. exsecta *and *F. pratensis*). However, in three other monogynous/weakly polygynous species (*F. rufa*, *F. lugubris*, *F. truncorum*) diploid males were found in fairly high frequencies, which indicates that diploid males are not necessarily an unbearable load.

### Social species without DMP

Unlike the non-social Hymenoptera there are no social Hymenoptera species for which the presence of sl-CSD has been refuted. Yet, there are some social Hymenopteran species known to inbreed consistently. Because inbreeding should result in DMP and no diploid males have yet been found in these species, these species are likely candidates for alternative mechanisms of sex determination. In the Japanese ant *Technomyrmex alpibes*, for example, incipient colonies produce wingless sexuals that inbreed for several generations [83]. For another Japanese ant, the harvesting ant, *Messor aciculatus*, genetic data revealed that mating swarms are drawn from very few colonies [84]. In the ant species *Cardiocondyla batesii *both sexes are flightless and Schrempf *et al*. [85] estimated that 83 per cent of the matings are between brothers and sisters. The social parasitic ants of the genus *Myrmoxenus *also have high levels of inbreeding, mating almost always occurs within the nest prior to dispersal [86].

## Genetical aspects of sl-CSD

### Evolution of thelytoky

Thelytokous species consist of only females that produce daughters parthenogenetically. Thelytoky occurs in all major groups of Hymenoptera, although it appears to be particularly abundant among the sawflies (Tenthredinoidea) and the parasitoid superfamilies Chalcidoidea and Cynipoidea. Thelytokous reproduction may be advantageous under certain environmental conditions and be of use in biological control [7]. Several authors have realised that sl-CSD may severely impair the evolution of thelytoky [87-90]. They implicitly assume that thelytoky evolved after sl-CSD. The reason is that several forms of thelytoky lead to an increase in homozygosity [91,92] and hence will yield diploid males rather than females in CSD species. Indeed, many species for which sl-CSD has been refuted (Table 3) belong to the superfamilies in which thelytoky is abundant.

Several forms of thelytoky are known from the Hymenoptera and each differs in its compatibility with sl-CSD (Table 4). The most extreme form is gamete duplication in which the meiotically produced haploid egg undergoes an extra round of DNA replication without cell division. The result is complete homozygosity. Gamete duplication has been demonstrated in only five species of Hymenoptera, two chalcidoids and three cynipoids (Table 4). Stouthamer and Kazmer [89] were the first to show that gamete duplication in *Trichogramma *was induced by cytoplasmically inherited *Wolbachia *bacteria. Such parthenogenesis inducing (PI) *Wolbachia *are now known from over 75 species of Hymenoptera (reviewed in [93]; Table 5). Sl-CSD is believed to be fully incompatible with *Wolbachia*-induced thelytoky. This mode of sex determination may therefore prevent the evolution of thelytoky by infection with PI-*Wolbachia*, this is supported by correlative taxonomic evidence. PI-*Wolbachia *are most abundant in the parasitoid superfamilies Chalcidoidea and Cynipoidea. These two groups appear to lack species with sl-CSD (Table 1). In contrast, PI-*Wolbachia *have not yet been found in the Tenthredinoidea (sawflies), Ichneumonoidea, Apoidea and Vespoidea. These are all groups in which sl-CSD is prevalent, and include the social Hymenoptera in which *Wolbachia *infection is over 50 per cent [94]. Even though thelytokous reproduction occurs among social Hymenoptera [95] it is has never been found to be caused by *Wolbachia*, this strongly suggest that sl-CSD has prevented the infection of PI-*Wolbachia *in the social Hymenoptera. For a number of groups (e.g. the sawflies), it is unclear how intensively they have been screened for *Wolbachia*, and there is a clear need for additional data on the link between sex determining mechanism and reproductive mode.

**Table 4:** Forms of thelytoky and their genetic effects.

Form of thelytoky	Genetic effect	Part of genome affected	Example	Reference
Premeiotic doubling	Fixed heterozygosity	All loci identical to mother	*Strongylogaster maculata* *Neoreterus baccarum* *Oecophylla longinoda* *Wasmannia auropunctata*	[177] [178,179] [180] [176]
Second division non-sister fusion	Increase in homozygosity at rate r^1^	Distal of crossing-overs	*Venturia canescens* *Apis mellifera capensis* *Cataglyphis cursor*	[181,182] [101,183,184] [103]
Second division sister fusion	Increase in homozygosity at rate 1–2r	Proximal of crossing-overs	*Diprion polytonum* *Pristiphora rufipes* *Aphytis mytilaspides*	[24] [185] [186]
Gamete duplication	Complete homozygosity	All loci	*Trichogramma spp.* *Muscidifurax uniraptor* *Diplolepis rosae* *Diplolepis spinosissimae* *Leptopilina clavipes*	[89] [96] [99] [98] [97]

^1 ^r = recombination rate, or map distance between a locus and its centromere [102].

**Table 5:** Number of thelytokous species and type of thelytoky for a number of Hymenopteran superfamilies

Hymenopteran superfamily	Number of thelytokous species^1^	Number of species with PI-*Wolbachia*^2^	Number of thelytokous species without PI-*Wolbachia*
Tenthredinoidea	90	0	3
Ichneumonoidea	32	5	1
Chalcidoidea	121	31	3
Cynipoidea	5^3^	16	3
Pelicinoidea	1	0	0
Proctotrupoidea	5	0	0
Bethyloidea	6	0	0
Apoidea	6	0	1
Vespoidea	10^4^	0	6

^1 ^= from [88], ^2 ^= from [93], ^3 ^= excluding >2000 species with cyclical thelytoky, ^4 ^= additional data from [65], [95], [103] and [176].

Caution needs to be exerted in extrapolating PI-*Wolbachia *to gamete duplication; for only five species has it been unequivocally demonstrated that gamete duplication is the mechanism by which thelytoky occurs in PI-*Wolbachia *infected species [89,96-99] see Table 4) and other mechanisms may occur [100]. There is a clear need for more cytological investigations of the mechanism of thelytoky in Hymenoptera in relation to sl-CSD and PI-*Wolbachia*.

Two other forms of thelytoky are fusion of second division sister and non-sister nuclei, also referred to as terminal and central fusion [92,101,102]. Here, either the two central polar nuclei of the second meiotic division fuse and form the egg from which the embryo develops, or the second polar nucleus fuses with the egg nucleus. Both processes lead to an increase in homozygosity over time, although they differ in the region of the genome that is affected. Under non-sister nuclei fusion, all loci distal of a cross-over have a 50 per cent chance of becoming homozygous depending on the segregation of the univalents during anaphase. Under sister nuclei fusion, proximal loci between the centromere and a cross-over have a 50 per cent chance of becoming homozygous. Both processes are therefore compatible with sl-CSD as long as the sex locus is located close to a centromere (non-sister fusion) or a telomere (sister fusion) respectively. Second division non-sister nuclei fusion has been reported in the honeybee (*Apis mellifera*) and a genetically similar mechanism in the ichneumonid *Venturia canescens *(Table 4). Another very special case is found in the ant *Cataglyphis cursor*. In this species, queens produce gynes predominantly by central fusion, while workers are produced by normal sexual reproduction [103]. As a result, the level of homozygosity is significantly higher in gynes than in workers, but no reports have been made of DMP. This suggests that this *C. cursor *either has an alternative sex determination system, or that the sex locus is located in a region of no recombination, such as close to a centromere or in an inversion. Fusion of second division nuclei has been found in two sawflies and the chalcidoid wasp *Aphytis mytilaspides *(Table 4). The sex locus in these species is expected to be located distally on one of the chromosomes.

Besides automictic (meiotic) parthenogenesis, apomixis (mitotic parthenogenesis) has been reported in four species of Hymenoptera; the sawfly *Strongylogaster maculata*, the cynipid *Neoretus baccarum *and the ant *Oecophylla longinoda *and the ant *Wasmannia auropunctata *(Table 4). Apomixis in these organisms occurs in the form of pre-meiotic doubling [92], this fixes heterozygosity and all offspring are identical to the mother. Pre-meiotic doubling is therefore fully compatible with sl-CSD because the heterozygous state of the sex locus in the female remains fixed.

### Other adaptations to CSD

In this section we discuss a number of known biological phenomena that may evolve in CSD species to overcome DMP. Although there is currently little evidence for most of these phenomena, this exercise is meant to draw attention to possible processes that have hitherto not been investigated, and to help to further focus future research in hymenopteran biology.

#### Evolution of more sex loci

One means of genetically reducing the risk of matched mating is to increase the number of sex loci, i.e. multi-locus CSD. Under the ml-CSD model (originally proposed by Snell [104] and extended by Crozier [105], there are two or more sex loci, each with multiple alleles, that determine sex. Heterozygosity at one or more loci is considered to result in females; only diploids that are homozygous at all loci will develop into males. Multi-locus CSD could evolve from single-locus CSD by a gene duplication event and a subsequent mutational change in the sex allele of one locus. Gene duplications are known to occur frequently during evolution [106]. However, thus far only once has ml-CSD been claimed to exist [107], in this study, the authors found evidence for two independent sex loci in the sawfly *Arge nigrinodosa*. At this moment it remains unclear whether ml-CSD occurs in more hymenopteran groups. Presence of ml-CSD has been rendered improbable for only two species based on prolonged inbreeding experiments [33,108] and for completely homozygous thelytokous wasps that have become sexual after *Wolbachia *removal [89]. More rigorous testing of species shown to lack sl-CSD is needed to determine the validity and prevalence of ml-CSD.

#### *Wolbachia *effects on sex determination

A theoretical possibility of how *Wolbachia *induced thelytoky could evolve in sl-CSD species is if the *Wolbachia *bacteria could overrule the hosts sex determining process, e.g. by making a product that turns diploid homozygous males into females. Although *Wolbachia *are known to affect several different developmental processes, including feminisation of genotypic males [109], a direct overruling of the sex determining process in haplodiploids has not yet been reported. However, it is not inconceivable given that (1) *Wolbachia *is widespread among Hymenoptera, (2) new effects of *Wolbachia *on their hosts are frequently discovered and (3) such an effect would provide a strong selective advantage to the micro-organism and may alleviate the diploid male load.

#### Selective fertilisation

Selective fertilisation is a well known phenomenon [110]; in many organisms females mate multiply and store sperm of several males. Sperm sorting refers to preferential fertilisation of eggs by particular types of sperm and implies sophisticated egg-sperm interactions. There is some evidence that eggs can gain information about the "content" of sperm through recognition of sperm surface proteins, before they make the "decision" of which of its own haplotypes will be lost in the second polar body [111,112]. In the case of sl-CSD, if eggs are able to recognise sperm with a matching sex allele and block fertilisation by such sperm, this would reduce or avoid the production of diploid male offspring in matched crosses. However, if females mate only once, they will receive only one type of sperm (due to haploidy of males). Theoretically, if females could recognise the sex allele in their eggs and control which eggs they fertilise, they could selectively fertilise those eggs that carry an unmatched allele and lay eggs with the matched allele as unfertilised males. Both sperm and egg sorting require the linkage of a signalling marker to the sex locus. Sperm selection has been proposed as an explanation for the deficiency of diploid males in natural populations of the communal bee *Andrena scotica *Perkins (= *A. jacobi*) [111]. These authors found that 44 per cent of all matings in this species were between sibs, whereas only 0.3 per cent of the diploids were male. Clearly, more attention needs to be paid to the possibility of selective fertilisation in Hymenoptera.

#### Selective self-ovicide

In some parasitoid species, females destroy the egg(s) of other females before they oviposit in the host themselves, a phenomenon known as ovicide [113] This behaviour probably evolved as a means of increasing the survival of their own eggs at the expense of eggs of conspecifics. It implies that females are able to distinguish their own eggs from those laid by other females. If females of CSD species were able to recognise matched eggs from unmatched eggs, selective self-ovicide of diploid male eggs would enable them to increase their reproductive output. Though self-ovicide has been reported for parasitoids [114] and recognition of diploid male brood is known in several social Hymenoptera (see above), recognition of diploid males and females at the egg stage has not been reported for Hymenoptera.

#### Viability and fertility of diploid males

Diploid males are frequently unviable, sterile or produce diploid sperm resulting in triploid sterile daughters (references in [31]). The recent work of Cowan and Stahlhut has challenged the view that such males are an evolutionary dead end. They reported evidence for normal fertility of diploid males in the wasp *Euodynerus foraminatus*. Their female offspring were diploid rather than triploid and inherited either one of the paternal marker alleles. At this time it is unclear by what mechanism diploidy of daughters is accomplished. Male hymenopterans have an abortive first meiotic division in spermatogenesis [102]. The authors suggest that diploid males may either produce haploid sperm by normal spermatogenesis or one chromosome set is eliminated from the fertilised egg. Selective elimination of a chromosome set during spermatogenesis [2] is another possibility. Whatever the mechanism may be, this study shows that selection could potentially also act to restore diploid male fertility by changes in the meiotic mechanism of spermatogenesis or in chromosome processing during the first mitotic division of the fertilised egg.

#### Matched genome inactivation

Paternal genome loss (PGL) exists in a number of mites and insects, including cynipid wasps, coccids and the fungal gnat *Sciara *[2], and has also been reported from the autoparasitoid *Encarsia pergandiella *[115]. In some forms of PGL males are effectively haploid because the paternally derived chromosomes are rendered inactive in male embryos through heterochromatisation and subsequent expulsion from the fertilised egg [2,74]. This process is believed to be under control of products put into the egg by the female. Theoretically, the disadvantages of DMP under sl-CSD could partly be overcome by the evolution of PGL. This would require females to be able to selectively eliminate the paternal genome if it carries a matched sex allele. Such females would produce fertile haploid males instead of sterile diploid males and although she would lose control over the sex ratio of her offspring, this could be selectively favourable in situations where the cost of producing males is not too high. Recognition of paternally and maternally inherited chromosome complements has been well documented, e.g. in the case of the Paternal Sex Ratio (PSR) chromosome [116], but the exact mechanisms are typically not known and neither are the conditions under which it evolved.

## Conclusions and outlooks for future research

This discussion highlights that there are still quite a number of intriguing questions to be answered before a full picture of the many genetical, ecological and evolutionary aspects of CSD becomes clear. We conclude this discussion by suggesting a number of research topics that, in our opinion, would contribute significantly to redressing this gap.

1. Diploid males have been reported in many more species than in which sl-CSD has actually been shown, notably in social Hymenoptera. In a few species, however, diploid males have been found that are not the result of sl-CSD, but rather that originated from mutation or hybridization. Thus, caution is required by directly inferring a role for sl-CSD from the presence of diploid males, and demonstrates the need to confirm CSD claims not only on the basis of DMP but also with molecular techniques or inbreeding experiments. Such experiments should carefully control for brood size, as diploid males may sometimes be unviable.

2. The taxonomic distribution of sl-CSD is still far from clear. Although there is a two-fold increase in the number of species suggested to have sl-CSD since previous reviews more than ten years ago [3,7,34], the information on the taxonomic distribution has increased to a much lesser extent, since many of these new species belong to the same taxa. We can only repeat Cook and Crozier's call to expand the search for CSD to other groups, most notably the Symphyta and allies, such as the primitive families Xyeloidea and Megalodontoidea. One key question of hymenopteran reproduction that can be resolved with this type of information is whether sl-CSD is indeed, as many researchers assume, the ancestral mode of sex determination.

3. In social Hymenoptera a number of special adaptations to CSD appear to have evolved, such as the elimination of diploid males and diploid male producing queens. In the non-social Hymenoptera, such as sawflies and solitary parasitoids some features of the oviposition behaviour can lead to a further temporal and spatial segregation of siblings. Whether these behaviours are specific adaptations to sl-CSD is not known. Groups of closely related species with and without sl-CSD such as the *Cotesia *genus (Table 1 and 3) offer good opportunities to study the adaptive significance of oviposition behaviour in relation to CSD. Such a comparative approach may reveal whether CSD imposes an important constraint on evolutionary processes in these species, or whether the sequential nature of oviposition itself is sufficient to overcome the disadvantages of sib mating under CSD.

4. In some groups alternative sex determining mechanisms apparently exist which provide escape from the disadvantages of sl-CSD. If sl-CSD is the ancestral mode, then these other mechanisms illustrate the evolutionary answers to the disadvantages of CSD. Studying these mechanisms, such as multi-locus CSD and diploid male fertility, is highly relevant in this respect, since they may act as possible stepping stones to undiscovered sex determining mechanisms in Hymenoptera. We have also discussed a number of hypothetical mechanisms that may have evolved to reduce the risk of diploid male production under sl-CSD. They include the evolution of multiple sex loci, selective fertilization, selective self-ovicide and matched genome inactivation. Attention should be given to these possibilities in future research on reproduction in Hymenoptera.

5. An increased understanding of the molecular genetic basis of sl-CSD will undoubtedly improve our insight into the restrictions placed upon species with this mode of sex determination. It will be very rewarding to reveal the mechanism of allelic complementation and how this process is modified in non-CSD species. In this respect, study of Braconidae may be particularly instructive because easy shifts between CSD and alternative mechanisms seem to occur. Furthermore, availability of whole genome sequences, such as for the honey bee and *Nasonia vitripennis*, will help in the identification of sex determining genes.

In conclusion, more than 65 years after the discovery of sl-CSD by Whiting [4] many questions remain unresolved. The study of CSD, however, remains highly relevant. For this mode of sex determination is likely to have played a major role in the evolution of most, if not all, groups of Hymenoptera. There are many economically important hymenopteran species, both beneficial and harmful, and an increased understanding of the genetical and ecological aspects of CSD will contribute to their culturing or control.
